# When the Cure Turns Into a Culprit: Mesalamine and the Eosinophilic Lung

**DOI:** 10.7759/cureus.116143

**Published:** 2026-09-12

**Authors:** Nithiyanandan Ravi, Bala Vigneshwar

**Affiliations:** 1 Pulmonary Medicine, Apollo Hospitals, Chennai, IND

**Keywords:** drug-induced lung disease, eosinophilic pneumonia, inflammatory bowel disease, mesalamine, pulmonary toxicity, steroids, ulcerative colitis

## Abstract

We report a case of a 74-year-old man with a one-year history of ulcerative colitis in remission who developed low-grade fever, dry cough, progressive dyspnea, loss of appetite, and an eight-kg weight loss over 12 weeks. Oral mesalamine (2.4 g/day) had been initiated 12 weeks before presentation, with respiratory symptoms developing approximately three weeks after initiation. Despite initial antibiotic therapy, his symptoms progressively worsened, and he developed hypoxemia requiring hospitalization. Investigations revealed leukocytosis with marked peripheral eosinophilia, elevated C-reactive protein (CRP) levels, and bilateral upper lobe- and peripheral-predominant pulmonary consolidations and ground-glass opacities. Infectious, parasitic, autoimmune, and malignant causes were investigated, and no alternative cause was identified. Transbronchial lung biopsy demonstrated eosinophil-rich subacute to chronic inflammation with organizing pneumonia-like changes, supporting eosinophilic pneumonia in the appropriate clinical context. The temporal relationship between mesalamine exposure and symptom onset, peripheral eosinophilia, compatible radiological and histopathological findings, and exclusion of alternative causes supported a diagnosis of mesalamine-associated eosinophilic pneumonia. Mesalamine was discontinued, and systemic corticosteroids were initiated concurrently, followed by rapid clinical improvement and subsequent complete radiological resolution. As both interventions were instituted simultaneously, the response could not be attributed independently to mesalamine withdrawal or corticosteroid therapy. This case highlights the importance of considering mesalamine-associated eosinophilic pneumonia in patients who develop respiratory symptoms and peripheral eosinophilia after mesalamine initiation. We also present a review of previously reported cases of 5-aminosalicylic acid (5-ASA)-associated eosinophilic respiratory disease.

## Introduction

Eosinophilic lung diseases are a rare and heterogeneous group of pulmonary disorders characterized by eosinophilic inflammation involving the lung parenchyma and, in some cases, the airways. They may be primary (idiopathic) or secondary to identifiable causes, including drugs, infections, environmental exposures, and systemic diseases [1]. Patients commonly present with fever, cough, dyspnea, and peripheral eosinophilia, while imaging may demonstrate bilateral peripheral consolidations or ground-glass opacities, often mimicking infectious pneumonia [2]. Mesalamine (5-aminosalicylic acid (5-ASA)) is widely used for the treatment of ulcerative colitis and is generally well tolerated. Although gastrointestinal and hypersensitivity reactions are more commonly reported, pulmonary toxicity, including eosinophilic pneumonia, is a rare but clinically important adverse effect [3]. The proposed mechanisms include immune-mediated hypersensitivity and direct pulmonary toxicity [4]. 

Diagnosis of mesalamine-associated eosinophilic pneumonia can be challenging because its clinical and radiological features overlap with infectious and parasitic infections, eosinophilic vasculitis, inflammatory bowel disease (IBD)- associated pulmonary disease, and malignancy. Although several cases of 5-ASA-associated eosinophilic respiratory disease have been reported, the evidence remains largely limited to individual case reports with variable clinical, radiological, and histopathological features.

We report a histopathologically confirmed case of mesalamine-associated eosinophilic pneumonia in a 74-year-old man with ulcerative colitis in remission who developed respiratory symptoms three weeks after mesalamine initiation. Notably, he was the oldest patient identified in our literature review. The case also highlights the diagnostic challenge posed by upper-lobe predominant consolidation and eosinophil-rich inflammation with organizing pneumonia-like changes on biopsy. We further review the reported 5-ASA-associated eosinophilic respiratory disease to characterize its clinical and diagnostic spectrum and emphasize the importance of considering drug-induced pulmonary toxicity when respiratory symptoms and eosinophilia develop following 5-ASA therapy.

## Case presentation

A 74-year-old man with a one-year history of ulcerative colitis, which was in remission, and comorbid diabetes mellitus, hypertension, and coronary artery disease presented to the outpatient department with low-grade fever, decreased appetite, an eight-kg weight loss over the preceding 12 weeks, dry cough, and progressive dyspnea (Modified Medical Research Council (MMRC) grade I progressing to grade II over three weeks) [5]. He denied any gastrointestinal symptoms. He had been started on oral mesalamine at a dose of 2.4 g/day 12 weeks before presentation, and his respiratory symptoms developed approximately three weeks after initiation of mesalamine.

On clinical examination, his vital signs were stable, and bilateral inspiratory crackles were heard on auscultation. A chest X-ray revealed right upper lobe consolidation (Figure 1A). He was initially treated with oral amoxicillin-clavulanate and doxycycline for five days for presumed infective pneumonia. However, his clinical condition worsened, and he was admitted to our inpatient department. On admission, he was hypoxemic, with a room-air oxygen saturation of 85%, and was started on oxygen supplementation through nasal prongs at 3 L/min. Baseline investigations revealed leukocytosis with peripheral eosinophilia, anemia, and elevated C-reactive protein (CRP) levels (Table 1).

**Figure 1:**
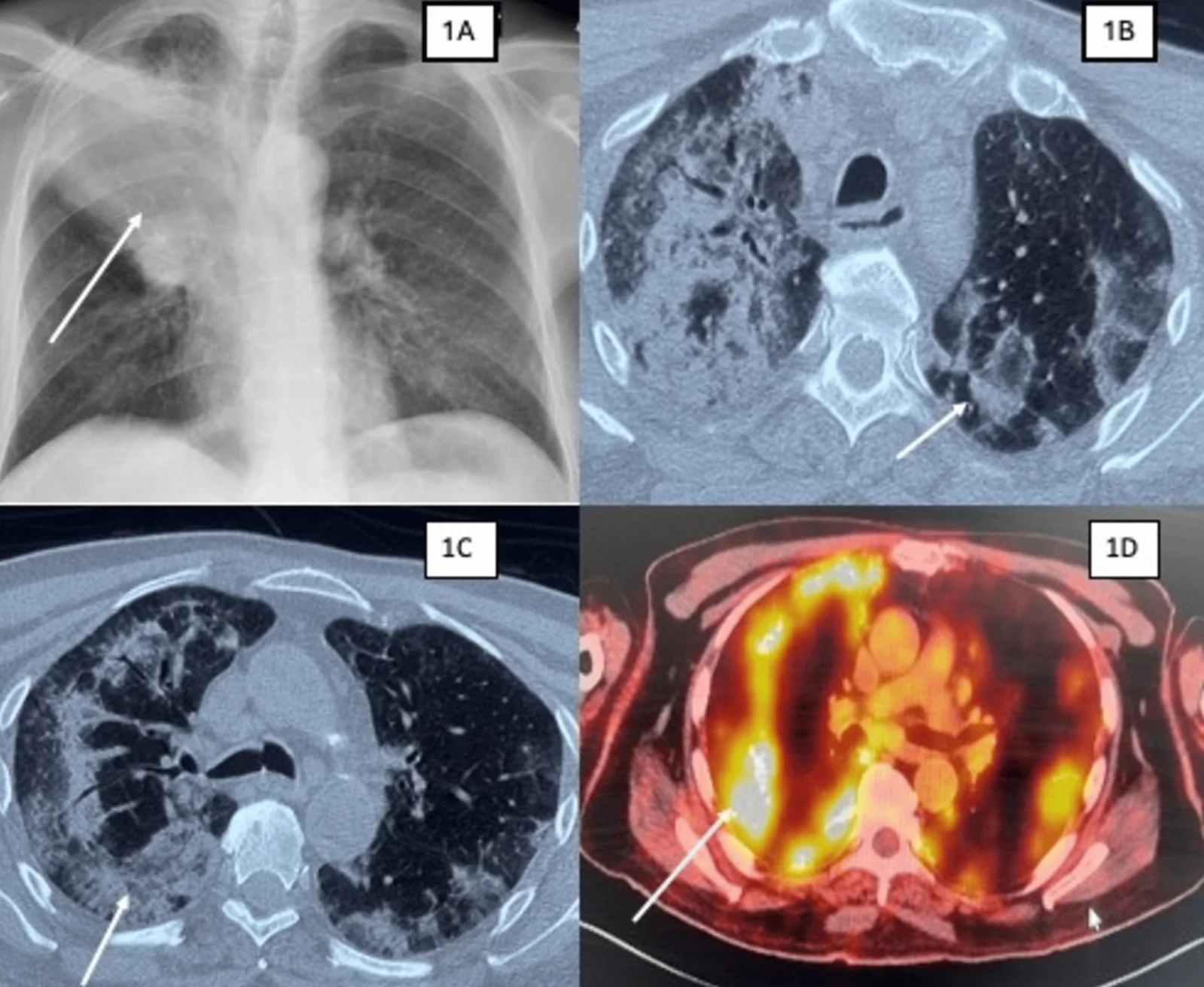
Baseline radiological images A: chest X-ray, PA view showing right upper lobe consolidation (white arrow); B, C: HRCT chest depicts right upper lobe-predominant bilateral patchy and peripheral consolidation with ground-glass opacities (white arrows); D: PET-CT showing FDG uptake in the areas of consolidation (white arrow). PA: posteroanterior; HRCT: high-resolution computed tomography; PET-CT: positron emission tomography-computed tomography; FDG: fluorodeoxyglucose

**Table 1:** Blood investigations The values show resolution of blood eosinophilia within two days following discontinuation of mesalamine and initiation of corticosteroids. CRP: C-reactive protein

Parameters	Before mesalamine	At symptom presentation (12 weeks after mesalamine initiation)	2 days after mesalamine withdrawal + corticosteroids	4-week follow-up	12-week follow-up
Hemoglobin (grams/dl)	13.9	10.2	8.7	12.1	12.9
Hematocrit (%)	43	32.5	28	38.8	39.5
WBC count (cells/mm^3^)	7690	14780	13840	10590	7850
Platelets (lakhs/mm^3^)	6.36	7.80	5.44	2.53	2.91
Neutrophils (%)	73	65	95	73	66.7
Lymphocytes (%)	15	8	3	20	17.5
Eosinophils (%)	1	23	0	2	4.7
Absolute eosinophil count (cells/mm^3^)	76	3400	0	212	368
Monocytes (%)	9	4	2	5	10.9
CRP levels (mg/L)		207.9		1	3

HRCT of the chest demonstrated bilateral, upper lobe- and peripheral-predominant patchy consolidations, ground-glass opacities, and interlobular septal thickening (Figures 1B, 1C). He was initially treated with intravenous cefoperazone-sulbactam, along with oral diethylcarbamazine and ivermectin. Investigations for alternative causes of pulmonary eosinophilia were negative, including an antinuclear antibody (ANA) profile by immunoblot, antineutrophil cytoplasmic antibody (ANCA) testing by enzyme-linked immunosorbent assay (ELISA) for anti-proteinase 3 (PR3) and anti-myeloperoxidase (MPO) antibodies, and stool examination for ova and cysts. Further evaluation revealed negative human immunodeficiency virus (HIV) serology, rheumatoid factor, and anti-cyclic citrullinated peptide (anti-CCP) antibodies.

Given the significant weight loss and persistent pulmonary consolidation despite initial antibiotic therapy, an underlying malignancy was considered, and whole-body PET-CT was performed to evaluate for malignancy. PET-CT demonstrated patchy irregular pulmonary opacities, ground-glass opacities, and interstitial thickening, predominantly involving the right upper lobe (Figure 1D).

Bronchoscopy with bronchoalveolar lavage (BAL) from the right upper lobe was negative for bacterial cultures, acid-fast bacilli (AFB) smear, fungal smear, *Nocardia* stain, and Xpert MTB/RIF. BAL cytology showed reactive cellular changes. Fluoroscopy-guided transbronchial lung biopsy from the right upper lobe revealed eosinophil-rich subacute to chronic inflammation with organizing pneumonia-like (bronchiolitis obliterans organizing pneumonia (BOOP)-like) changes (Figure 2 shows eosinophilic infiltration of lung parenchyma). Although organizing pneumonia-like changes were present, the prominent eosinophilic inflammatory component, together with peripheral blood eosinophilia and the characteristic clinical and radiological findings, supported eosinophilic pneumonia rather than primary organizing pneumonia.

**Figure 2:**
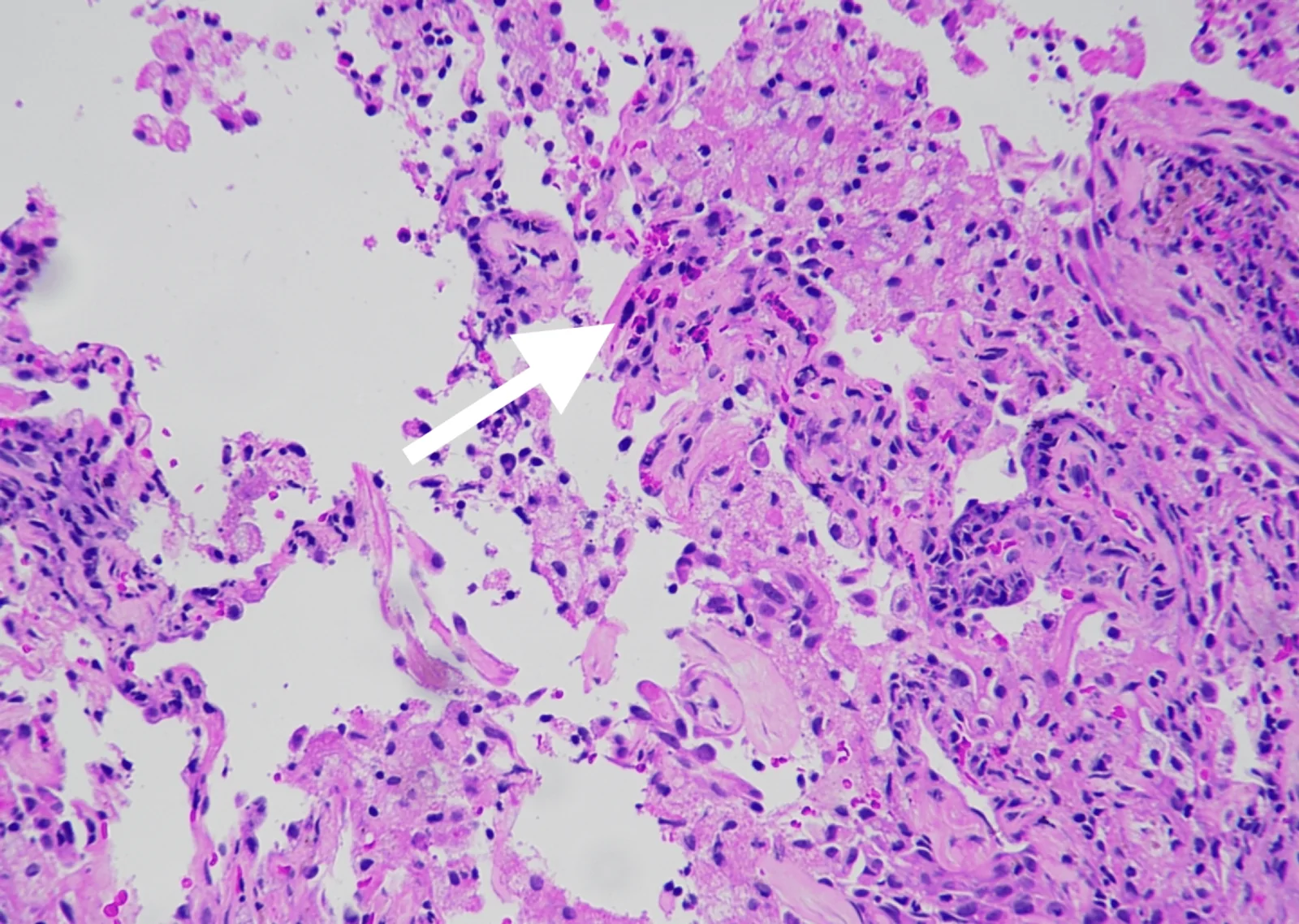
Histopathology from transbronchial lung biopsy Lung biopsy (H&E stain, high power) demonstrating prominent eosinophilic infiltration (white arrow) of the interstitium and alveolar spaces, with alveolar wall thickening.

In view of the onset of respiratory symptoms three weeks after initiation of mesalamine, peripheral eosinophilia, characteristic radiological findings, exclusion of infectious, autoimmune, and malignant causes, and eosinophil-rich inflammation on transbronchial lung biopsy, mesalamine-associated eosinophilic pneumonia was considered. Mesalamine was discontinued, and intravenous methylprednisolone 40 mg twice daily was initiated concurrently. Within two days, the patient showed marked clinical improvement, with resolution of hypoxemia and normalization of the eosinophil count (Table 1). As mesalamine withdrawal and corticosteroid therapy were instituted concurrently, the clinical response could not be attributed to either intervention individually. He was discharged on oral prednisolone at 0.5 mg/kg/day.

At four-week follow-up, he remained afebrile, with significant improvement in cough and dyspnea. His appetite improved; he had gained two kg in weight, and a chest X-ray showed complete resolution of the consolidation (Figure 3A). Corticosteroids were gradually tapered and discontinued after 10 weeks. At 12-week follow-up, he had gained a total of 10 kg, and HRCT of the chest showed complete resolution of the pulmonary consolidations (Figure 3B). Considering the temporal relationship with mesalamine exposure, peripheral eosinophilia, compatible radiological findings, eosinophil-rich pulmonary inflammation on biopsy, exclusion of alternative causes, and subsequent clinical and radiological improvement following mesalamine discontinuation with corticosteroid therapy, a diagnosis of probable mesalamine-associated eosinophilic pneumonia was made.

**Figure 3:**
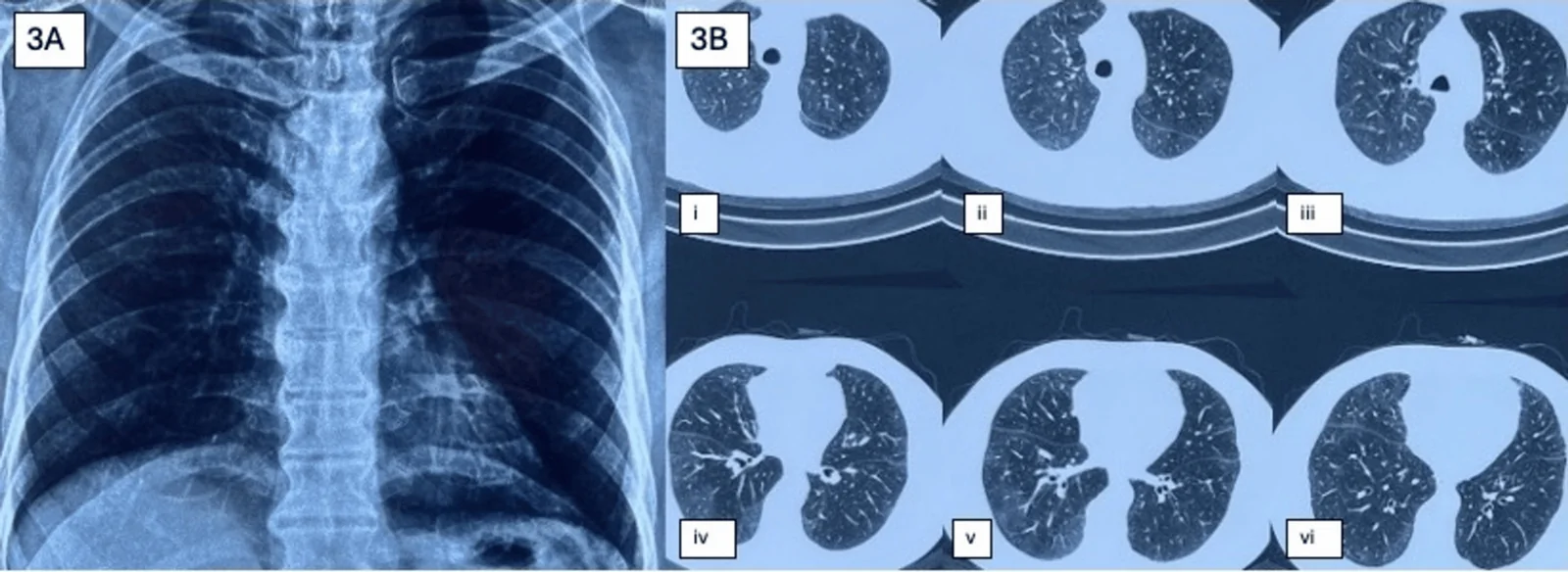
Follow-up radiological images A: follow-up chest X-ray done at four weeks (four weeks after stopping mesalamine and starting systemic steroids), showing complete resolution of opacities; B: follow-up HRCT chest images (i, ii, iii, iv, v, vi) done at 12 weeks (12 weeks after stopping mesalamine and starting systemic steroids), showing complete resolution of opacities.

## Discussion

In our index case, the differential diagnoses included infectious pneumonia, parasitic infection, eosinophilic vasculitis, IBD-associated pulmonary disease, malignancy, and mesalamine-induced eosinophilic lung disease. Infectious pneumonia was considered initially; however, the patient worsened despite antibiotics, and BAL was negative for bacterial, fungal, mycobacterial, and *Nocardia* infections, including Xpert MTB/RIF. Parasitic infection was considered because of pulmonary eosinophilia but was less likely given the negative stool examination and absence of other evidence of parasitic disease. Eosinophilic vasculitis was considered because of peripheral eosinophilia, but ANA and ANCA testing was negative, and there were no systemic features of vasculitis. IBD-associated pulmonary disease was also considered; however, the ulcerative colitis was in remission, there were no gastrointestinal symptoms, and the marked eosinophilia, eosinophil-rich biopsy findings, and temporal association with mesalamine favored drug-induced disease. Malignancy was considered because of the eight-kg weight loss and persistent consolidation; whole-body PET-CT did not demonstrate a definite primary or metastatic malignancy. 

Drug-induced eosinophilic pneumonia should be considered when five criteria are fulfilled: no other likely cause of lung disease; symptoms consistent with the suspected drug; a time course compatible with drug-induced lung disease; tissue or BAL findings compatible with drug-induced lung disease; and improvement following discontinuation of the suspected drug. Patients fulfilling all five criteria are considered to have definite drug-induced eosinophilic lung disease, those fulfilling four criteria are considered to have probable disease, and those fulfilling three criteria are considered to have suspected disease [6]. In our patient, these criteria were supported by the negative evaluation for alternative causes, compatible respiratory manifestations, onset approximately three weeks after mesalamine initiation, eosinophil-rich inflammation on lung biopsy, and clinical and radiological improvement following mesalamine withdrawal with corticosteroid therapy. However, because corticosteroids were initiated concurrently, the improvement cannot be attributed specifically to mesalamine withdrawal. Thus, the criteria support rather than independently establish drug causality.

The Naranjo algorithm is commonly used to assess the probability of an adverse drug reaction (ADR) [7]. Our patient had a Naranjo score of 7, indicating a probable ADR (Table 2). However, the Naranjo score is supportive rather than definitive evidence of causality, particularly in drug-induced pulmonary disease. Rechallenge was not performed because of the potential risk of recurrent pulmonary toxicity, and corticosteroids were started concurrently with mesalamine withdrawal. Therefore, the treatment response could not be attributed exclusively to drug withdrawal. The Naranjo score was consequently interpreted together with the clinical, radiological, laboratory, histopathological, and temporal findings rather than as independent proof of mesalamine-associated pulmonary toxicity. 

**Table 2:** Naranjo adverse drug reaction probability scale in the index case IBD: inflammatory bowel disease

No.	Naranjo criterion	Finding in our patient	Response	Score
1	Are there previous conclusive reports on this reaction?	Previous published cases of mesalamine-associated eosinophilic pulmonary disease are reported in the literature.	Yes	+1
2	Did the adverse event appear after the suspected drug was administered?	Respiratory symptoms developed approximately 3 weeks after initiation of mesalamine 2.4 g/day.	Yes	+2
3	Did the adverse reaction improve when the suspected drug was discontinued?	Clinical improvement, resolution of hypoxemia, normalization of eosinophil count, and subsequent radiological resolution occurred after mesalamine withdrawal and corticosteroid therapy.	Yes	+1
4	Did the adverse reaction reappear when the drug was readministered?	Mesalamine was not rechallenged because of the risk of recurrent pulmonary toxicity.	Do not know	0
5	Are there alternative causes that could have caused the reaction?	Infectious, parasitic, autoimmune/vasculitic, IBD-related, and malignant causes were investigated, with no alternative cause identified.	No	+2
6	Did the reaction reappear when a placebo was given?	No placebo challenge was performed.	Do not know	0
7	Was the drug detected in blood or other fluids at toxic concentrations?	No drug concentration measurement was performed.	Do not know	0
8	Was the reaction more severe with dose increase or less severe with dose decrease?	No dose-response relationship was demonstrated in this patient.	Do not know	0
9	Did the patient have a similar reaction to the same or similar drug during previous exposure?	No previous similar reaction was documented.	Do not know	0
10	Was the adverse reaction confirmed by objective evidence?	Peripheral eosinophilia, characteristic HRCT findings, and transbronchial lung biopsy showing eosinophil-rich inflammation with organizing pneumonia-like changes provided objective evidence.	Yes	+1
	Total score			7

Mesalamine-related pulmonary toxicity encompasses eosinophilic pneumonia, BOOP, granulomatous interstitial lung disease (ILD), lymphocytic pneumonitis, interstitial fibrosis, interstitial pneumonitis, eosinophilic pleural effusion, and bronchospasm, with proposed mechanisms including immunological reactions, direct pulmonary toxicity, oxidant injury, and intracellular phospholipid deposition [8]. IBD can cause diverse pulmonary manifestations, including airway disease, ILD, pleural disease, and necrobiotic lung nodules, making differentiation from mesalamine-induced lung injury challenging [9,10]. Because both IBD and mesalamine can produce pulmonary parenchymal and airway abnormalities, the distinction should be based on the overall clinical, radiological, laboratory, histopathological, and temporal findings. In our patient, the remission of IBD, absence of gastrointestinal symptoms, prominent peripheral eosinophilia, compatible imaging, eosinophil-rich biopsy, and temporal relationship to mesalamine favored drug-induced eosinophilic pneumonia. A drug-induced lymphocyte stimulation test has limited sensitivity, and a negative result does not exclude mesalamine-induced pneumonitis [11]. The biopsy demonstrated eosinophil-rich subacute to chronic inflammation with organizing pneumonia-like (BOOP-like) changes. Although organizing pneumonia and eosinophilic pneumonia are distinct pathological patterns, they may overlap. In this case, the prominent eosinophilic component, peripheral eosinophilia, and compatible radiological findings supported eosinophilic pneumonia rather than primary organizing pneumonia [12]. 

A literature search was conducted in August 2026 using PubMed with the MeSH terms (“Mesalamine”[Mesh]) AND (“Pulmonary Eosinophilia”[Mesh]), supplemented by a Google search using the terms “mesalamine and eosinophilic pneumonia.” English-language case reports describing eosinophilic respiratory disease associated with mesalamine or other 5-ASA agents were considered eligible. Titles and abstracts were screened, relevant full-text articles were reviewed, and reference lists of eligible reports were manually searched for additional cases. Reports without sufficient clinical information and non-English publications were excluded. Including our index case, 28 cases were identified: 27 with eosinophilic pneumonia and one with eosinophilic bronchitis (Table 3). Sulfasalazine and balsalazide cases were retained as 5-ASA-associated disease but were separately identified from mesalamine cases in Table 3.

**Table 3:** 5-ASA-associated eosinophilic respiratory diseases (summary of reported cases) 5-ASA: 5-aminosalicylic acid; AEC: absolute eosinophil count; US: ulcerative colitis; CD: Crohns disease; VATS: video-assisted thoracoscopic surgery; F: fever; C: cough; D: dyspnea; WL: weight loss; LOA: loss of appetite; H: hypoxia; M: malaise; R: skin rash; NA: not available in study; BAL: bronchoalveolar lavage; TBLB: transbronchial lung biopsy; B/L: bilateral; GGOs: ground-glass opacities; GI: granulomatous inflammation; UL: upper lobe; RML: right middle lobe; RLL: right lower lobe; RUL: right upper lobe; CXR: chest X-ray; CT: computed tomography; FENO: fractional exhaled nitric oxide; *: possible mesalamine-azathioprine synergism; $: mesalamine induced eosinophilic bronchitis

Author	Year	Age/gender	IBD	5-ASA agent	Dosage/day	Interval	Clinical features	AEC (highest) count/mm^3^	Radiology	Method of diagnosis	Treatment	Outcome
Sullivan et al. [13]	1987	40/M	UC	Sulfasalazine	6g	8 weeks	F, C, D, WL, H	2100	CXR: B/L UL infiltrates	Open lung biopsy	Stopped sulfasalazine	Full recovery
Honeybourne et al. [14]	1994	30/F	UC	Mesalamine	1.6g	7 months	F, C, D	High (16%)	NA	TBLB	Stopped mesalamine	Full recovery
Tanigawa et al. [15]	1999	35/F	UC	Mesalamine	1.5g	40 days	F, C	164	CXR and CT chest: B/L and peripheral filtrates	TBLB and positive drug lymphocyte stimulation test	Stopped mesalamine	Full recovery
Saltzman et al. [16]	2001	53/F	UC	Mesalamine	NA	4 months	F, C, M, R	2200	CXR: Right-sided infiltrates	BAL and TBLB	Stopped mesalamine, started prednisolone 60mg/day	Full recovery
Katsenos et al. [4]	2007	18/M	UC	Mesalamine^*^+ azathioprine	NA	1 year	F, C	700	CXR and CT chest: B/L UL predominant infiltrates, more in left UL	BAL: Eosinophilia, TBLB: Non-diagnostic	Stopped mesalamine, started prednisolone 40mg/day	Full recovery
Patel et al. [17]	2007	16/F	UC	Mesalamine	NA	2 months	F, C, D, WL, night sweats, chills	Increased	CXR and CT chest: B/L UL and middle lobe peripheral opacities	Clinical and radiological	Stopped mesalamine and started steroids	Full recovery
Price et al. [18]	2007	28/F	UC	Mesalamine	1.2g	5 years	C, F, wheeze, WL, nasal congestion	2000	CT chest: RML and RLL bronchiectasis, RLL nodules	Clinical and radiological; BAL: No eosinophilia	Stopped mesalamine and started prednisolone 25mg	Full recovery
Kumar et al. [19]	2008	28/F	UC	Balsalazide + Mesalamine	4.8g (molar equivalent dose of mesalamine)	Several months	C, D	NA	CT chest: Lobar consolidation, nodular infiltrates	TBLB: Eosinophilia, Non-necrotizing GI seen	Stopped mesalamine	Full recovery
Fayaz et al. [20]	2009	24/F	UC	Mesalamine	NA	2 years	D, chest pain	Increased	B/L infiltrates and left-sided pleural effusion	Thoracoscopy and pleural biopsy	Stopped mesalamine	Full recovery
McGrane et al. [21]	2010	44/F	UC	Balsalazide	NA	1 week	C, D, WL, lethargy, night sweats	8380	CT chest: Left-sided centrilobular nodules, GGOs	TBLB	Stopped balsalazide	Full recovery
Kim et al. [8]	2013	30/F	UC	Mesalamine suppository	1 g intrarectal	19 days	C	504	CT chest: B/L patchy consolidation and ST	TBLB	Stopped mesalamine, started steroids	Full recovery
Inoue et al. [11]	2014	15/F	UC	Mesalamine	2.25g	4 weeks	C	1100	CT chest: B/L patchy consolidation	Clinical, radiological, and positive drug-induced lymphocyte stimulation test	Stopped mesalamine	Developed pneumothorax; treated with chest drain and achieved full recovery
Inoue et al. [11]	2014	48/F	UC	Mesalamine	NA	NA	D	9600	CT chest: B/L peripheral consolidation (right side dominated)	Drug-induced lymphocyte stimulation test: Negative; BAL and TBLB suggestive	Stopped mesalamine	Full recovery
Inoue et al. [11]	2014	50/F	UC	Mesalamine	3.6 g (1^st^ 2 weeks), 2.4 g (from 3^rd^ week)	5 weeks	F, C	4300	CT chest: B/L patchy consolidation (upper lobe predominant)	Clinical and radiological: Drug-induced lymphocyte stimulation test: negative	Stopped mesalamine	Full recovery
Ferrusquia et al. [22]	2015	32/M	UC	Mesalamine	1.5g (increased to 3g last 3 weeks)	14 months	F, C, D, asthenia, night sweats, chest pain	7800	CT chest: B/L centrilobular nodules, GGOs, pericardial effusion and mediastinal lymphadenopathy	BAL and TBLB	Stopped mesalamine and started steroids	Full recovery
Franco et al. [23]	2016	39/F	UC	Mesalamine	3.6g	2 months	F, C, D, H, night sweats	807	CT chest: B/L infiltrates	BAL and TBLB	Stopped mesalamine and started steroids	Full recovery
Zhang et al. [24]	2016	56/F	UC	Mesalamine	1.5g	6 weeks	F, C, D, chest pain, fatigue	879	CT chest: B/L upper lobe predominant GGOs and consolidation	BAL: Eosinophilia; TBLB: Not suggestive	Stopped mesalamine	Full recovery
Gupta et al. [25]	2017	22/M	UC	Mesalamine	NA	6 months	F, C, wheezing, H	NA	CT chest: B/L patchy GGOs	BAL: Eosinophilia	Stopped mesalamine and started steroids	Full recovery
Bonarigo et al. [26] (Abstract)	2017	27/M	UC	Mesalamine	1.2g	1.5 years	F, C, D, WL	1000	CT chest: B/L UL peripheral opacities	TBLB	Stopped mesalamine and started steroids	Full recovery
Yeo et al. [27]	2017	26/M	CD	Mesalamine	3g	10 months	C, D, chest discomfort	519	CXR, CT chest: B/L subpleural consolidation and GGOs	BAL: Eosinophils 14%, FENO:74 ppb	Stopped mesalamine and started prednisolone 30mg/day	Full recovery, FENO reduced to 13 ppb
Park et al. [28]	2018	35/F	UC	Mesalamine	1g	1 month	F, C, D	1098	CT chest: B/L multifocal consolidation	VATS wedge resection	Stopped mesalamine and started steroids (MP 500 mg/d for 3 days, then prednisolone 40mg/day	Full recovery
Fehily et al. [29]	2019	27/F	UC	Mesalamine	4.8g	18 months	F, C, D	4700	CT chest: B/L upper lobe consolidation and GGOs, right-sided pleural effusion	BAL and TBLB	Stopped mesalamine and started prednisolone 25 mg/day	Full recovery
Li et al. [30]	2024	24/F	UC	Mesalamine	NA	10 days	C	1620	CT chest: B/L patchy opacities	CT-guided lung biopsy	Stopped mesalamine and started prednisolone 40mg/day	Full recovery; recurred after mesalamine was restarted
Rajendram et al. [31]	2025	71/F	UC	Mesalamine	4g	NA	D, C, fatigue, H, LOA, WL, orthopnea, peripheral edema	Elevated	CT chest: B/L subpleural interstitial thickening with GGOs	Clinical	Stopped mesalamine, started steroids	Full recovery
Sharma et al. [32]	2025	27/M	UC	Mesalamine	NA	3 months	C, fatigue, chest tightness	NA	CT chest: Left UL infiltrates	Biopsy	Stopped mesalamine	Full recovery
Nasim et al. [33]	2026	24/M	UC	Mesalamine: oral plus suppository	4g	2 years and 4 months	C, D	4900	CT chest: B/L subpleural GGOs and consolidation	Clinical	Stopped mesalamine and started steroids	Full recovery
Index case	2026	74/M	UC	Mesalamine	2.4g	3 weeks	F, C, D, H, WL, LOA	3400	CT chest: B/L patchy consolidation with predominant RUL consolidation	BAL plus TBLB	Stopped mesalamine and started steroids	Full recovery (at 4 weeks follow up)
Cernomaz et al. [34]^$^	2020	40/F	UC	Mesalamine	6g	2 weeks	C, mild hemoptysis	780	CT chest: Irregular circumferential thickening of subglottic region and upper trachea	Tracheal and bronchial mucosal biopsy: Eosinophilic bronchitis	Stopped mesalamine and started inhaled steroids and bronchodilator	Full recovery

The offending agents were mesalamine alone in 22 cases, mesalamine with azathioprine in one case, mesalamine suppository alone in one case, oral mesalamine with mesalamine suppository in one case, mesalamine with balsalazide in one case, sulfasalazine alone in one case, and balsalazide alone in one case. Thus, mesalamine was implicated in 26 of the 28 cases, either alone or in combination, whereas two cases involved other 5-ASA agents without mesalamine (one sulfasalazine and one balsalazide). Most patients had ulcerative colitis (27/28, 96.4%), with only one patient having Crohn’s disease. There was a female predominance (19/28, 67.9%), and the age of patients ranged from 15 to 74 years. Reported doses ranged from 1 to 6 g/day, while the duration of exposure varied widely. However, these case reports are insufficient to establish a dose-response or duration-response relationship because of reporting bias, heterogeneous case ascertainment, and the absence of a denominator of exposed patients.

Among the 27 cases of eosinophilic pneumonia, cough (25/27, 92.6%), dyspnea (17/27, 63.0%), and fever (16/27, 59.3%) were the most common symptoms, followed by weight loss (25.9%), chest pain or discomfort (18.5%), fatigue (18.5%), hypoxia (18.5%), night sweats (14.8%), and wheezing (7.4%). Peripheral eosinophilia was reported in most cases, with absolute eosinophil count (AEC) values ranging from 164 to 9600 cells/mm³. Radiological findings most commonly included bilateral peripheral or upper-lobe predominant consolidation and ground-glass opacities, while nodules, interstitial infiltrates, bronchiectasis, pleural effusion, and unilateral opacities were less frequent. Diagnosis was primarily based on lung biopsy and/or BAL demonstrating eosinophilic infiltration, although some cases were diagnosed on compatible clinical and radiological findings. Drug-induced lymphocyte stimulation tests were inconsistently performed, with both positive and negative results, limiting their diagnostic utility.

Reported outcomes were generally favorable following discontinuation of the offending drug. Systemic corticosteroids were used in most cases, although the available case reports do not allow the independent effect of corticosteroid therapy to be determined. The single case of eosinophilic bronchitis presented with cough and mild hemoptysis; CT demonstrated tracheobronchial thickening, and tracheal mucosal biopsy confirmed eosinophilic bronchitis. The patient recovered following mesalamine withdrawal, inhaled corticosteroids, and bronchodilator therapy.

## Conclusions

Mesalamine-associated eosinophilic pulmonary disease is a rare but important adverse effect that should be considered when patients develop new respiratory symptoms and peripheral eosinophilia after initiation of mesalamine. The combination of clinical, radiological, and histopathological findings, together with exclusion of alternative causes and improvement following mesalamine withdrawal and corticosteroid therapy, supports a probable association with mesalamine in the present case. Early recognition and discontinuation of mesalamine are central to management, with systemic corticosteroids considered according to clinical severity. Reported cases generally describe favorable outcomes; however, the available evidence is based predominantly on case reports and therefore cannot establish definitive causality, incidence, or a predictable treatment response. Rare airway manifestations, such as eosinophilic bronchitis, may also occur.
